# Longitudinal profiling of clonal hematopoiesis provides insight into clonal dynamics

**DOI:** 10.1186/s12979-022-00278-9

**Published:** 2022-05-24

**Authors:** Md Mesbah Uddin, Ying Zhou, Alexander G. Bick, Bala Bharathi Burugula, Siddhartha Jaiswal, Pinkal Desai, Michael C. Honigberg, Shelly-Ann Love, Ana Barac, Kathleen M. Hayden, JoAnn E. Manson, Eric A. Whitsel, Charles Kooperberg, Pradeep Natarajan, Alexander P. Reiner, Jacob O. Kitzman

**Affiliations:** 1 Cardiovascular Research Center, Massachusetts General Hospital, Boston, MA USA; 2 Program in Medical and Population Genetics and the Cardiovascular Disease Initiative, Broad Institute of Harvard and MIT, Cambridge, MA USA; 3 Division of Public Health Sciences, Fred Hutchinson Cancer Center, Seattle, WA USA; 4 Division of Genetic Medicine, Department of Medicine, Vanderbilt University Medical Center, Nashville, TN USA; 5 Department of Human Genetics, University of Michigan, Ann Arbor, MI USA; 6 Department of Pathology, Stanford University School of Medicine, Stanford, CA USA; 7 Weill Cornell Medicine, New York, NY USA; 8 Department of Medicine, Harvard Medical School, Boston, MA USA; 9 Department of Epidemiology, University of North Carolina, Gillings School of Global Public Health, Chapel Hill, NC USA; 10 Department of Cardiology, MedStar Heart and Vascular Institute, Georgetown University, Washington, DC USA; 11 Wake Forest University School of Medicine, Winston-Salem, NC USA; 12 Department of Medicine, Brigham and Women’s Hospital, Harvard Medical School, Boston, MA USA; 13 Department of Epidemiology, University of Washington, Seattle, WA 98109 USA

**Keywords:** Clonal hematopoiesis, Somatic mutations, Longitudinal analysis, Aging

## Abstract

**Background:**

Clonal hematopoiesis of indeterminate potential (CHIP), the age-related expansion of mutant hematopoietic stem cells, confers risk for multiple diseases of aging including hematologic cancer and cardiovascular disease. Whole-exome or genome sequencing can detect CHIP, but due to those assays’ high cost, most population studies have been cross-sectional, sequencing only a single timepoint per individual.

**Results:**

We developed and validated a cost-effective single molecule molecular inversion probe sequencing (smMIPS) assay for detecting CHIP, targeting the 11 most frequently mutated genes in CHIP along with 4 recurrent mutational hotspots. We sequenced 548 multi-timepoint samples collected from 182 participants in the Women’s Health Initiative cohort, across a median span of 16 years. We detected 178 driver mutations reaching variant allele frequency ≥ 2% in at least one timepoint, many of which were detectable well below this threshold at earlier timepoints. The majority of clonal mutations (52.1%) expanded over time (with a median doubling period of 7.43 years), with the others remaining static or decreasing in size in the absence of any cytotoxic therapy.

**Conclusions:**

Targeted smMIPS sequencing can sensitively measure clonal dynamics in CHIP. Mutations that reached the conventional threshold for CHIP (2% frequency) tended to continue growing, indicating that after CHIP is acquired, it is generally not lost. The ability to cost-effectively profile CHIP longitudinally will enable future studies to investigate why some CHIP clones expand, and how their dynamics relate to health outcomes at a biobank scale.

**Supplementary Information:**

The online version contains supplementary material available at 10.1186/s12979-022-00278-9.

## Background

Chronological age is the dominant risk factor for cancers and cardiovascular disease – the leading causes of death worldwide [1]. Aging is also associated with a higher prevalence of acquired somatic mutations, especially in frequently regenerating cells, such as hematopoietic stem cells (HSC). Clonal hematopoiesis of indeterminate potential (CHIP) is the age-related expansion (defined as variant allele fraction, VAF ≥2%) of cancer-associated somatic mutations (typically in *DNMT3A*, *TET2*, *ASXL1*, *JAK2*) in hematopoietic stem cells in the absence of unexplained cytopenia, dysplasia, or neoplasia [2]. Recent whole exome sequence (WES) and whole genome sequence (WGS) analyses of blood-derived DNA have shown that CHIP is increasingly common with advancing age (i.e., approximately 10% of asymptomatic adults older than 70 years of age) [3–6]. While CHIP is a risk factor for hematologic malignancy and all-cause mortality [3, 7, 8], a number of analyses have shown an association with atherosclerotic cardiovascular disease [4, 9, 10]. CHIP is also associated with heightened risk of therapy-related myeloid malignancies [11–14]. These studies underline the importance of CHIP as a novel biomarker for early detection and monitoring of multiple age-related diseases [15, 16]. However, further longitudinal studies are needed for a better understanding of the root causes of CHIP, surveillance strategies, and how CHIP dynamics influence the development of chronic diseases.

Sensitivity for the detection of driver mutations is highly dependent on sequencing depth. Both WGS and WES are suitable for the detection of larger clones (e.g., VAF > 5% in WGS [6, 7], and VAF > 3% in WES [3, 4]). By comparison, deeper coverage, error-corrected targeted sequencing techniques are capable of detecting very small clones [8, 15], which are nearly ubiquitous in healthy adults [17]. Additional studies of apparently healthy adults characterizing longitudinal changes in clone size over time may reveal genetic and environmental factors promoting clonal stability versus progression and yield new insights into mechanisms underlying somatic mutagenesis and aging as well as resultant disease pathogenesis and disease prediction.

Here we present a single-molecule molecular inversion probe sequencing (smMIPS) assay [18], that leverages a cost-effective, ultrasensitive, high-throughput targeted sequencing technique, for the detection of CHIP. We apply this assay to a set of longitudinal peripheral blood DNA samples obtained over a median range of 16 years from 182 post-menopausal women from the Women’s Health Initiative to compare to whole genome sequence analysis and evaluate clonal dynamics.

## Results

### CHIP panel design and assay validation

**Fig. 1:**
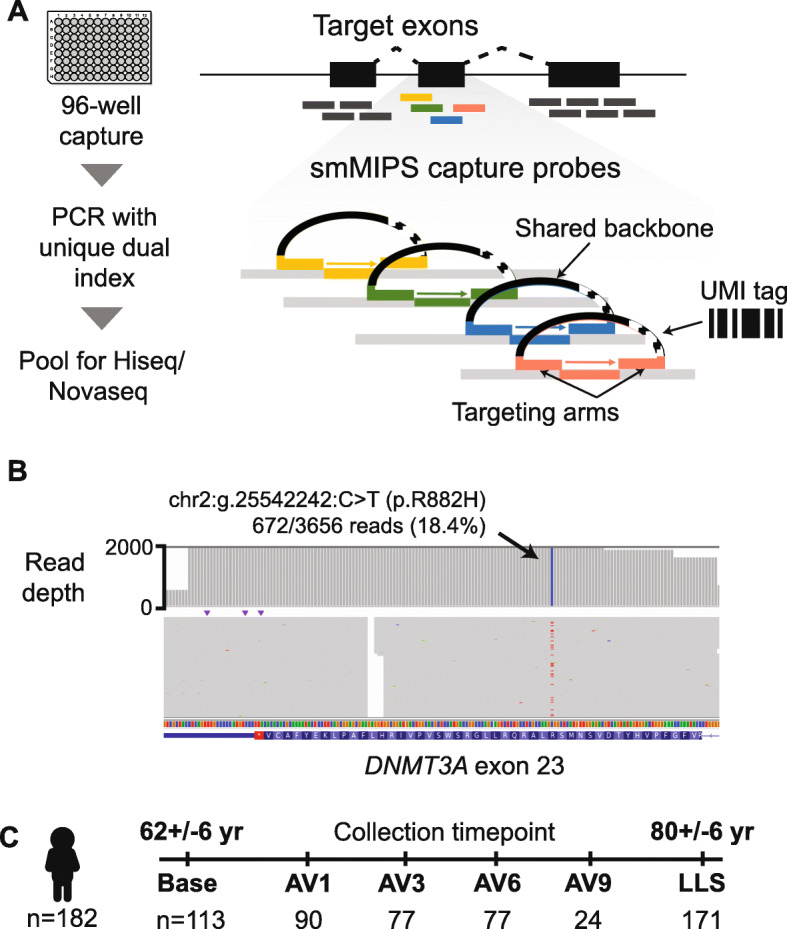
Study design and CHIP sequencing strategy. **A** Schematic of smMIPS assay design. **B** Somatic mutation identified as CHIP by smMIPS assay. **C** Schematic of study design, with sequencing of each subject (*n* = 182) using samples collected at up to six timepoints including a baseline visit, a series of annual visits (AV), and a final visit (LLS, Long Life Study)

**Fig. 2:**
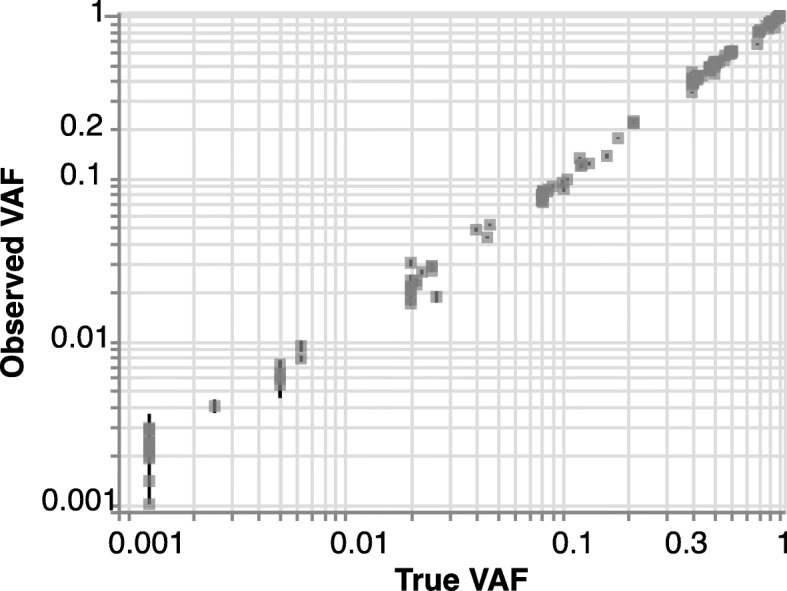
Validation by sequencing defined sample mixtures. Observed (mean +/− s.e. across 27 replicates) vs expected variant allele frequency (VAF) for repeated smMIPS sequencing of a defined control mixture of gDNAs from five cell lines, across 152 polymorphic sites. Overall Pearson’s correlation *r* = 0.998, and *r* = 0.847 and *r* = 0.997 for variants with expected VAF ≤2 and > 2%, respectively

We designed a smMIPS capture panel tiling all coding exons (±5 bp) across the 11 most common CHIP genes, along with mutational hotspots in four other genes (Fig. 1A and Table 1). The final capture included 3526 probes, each containing a 9-mer unique molecular index (UMI) for duplicate read removal, spanning a total of 35.2 kb of genomic sequence in the target region. To validate this panel, we first re-sequenced five HapMap lymphoblastoid cell lines (LCLs) and successfully identified all variants defined by 1000G WGS datasets in the target region (*n* = 152), with no additional variants called. Focusing on positions invariant in these cell lines, we estimate a low sequencing error rate of 0.045% (~ 1/2200 bp; Supplementary Note). Next, to mimic driver mutations across a range of variant allele fractions (VAF) starting well below the conventional CHIP threshold of 2%, we mixed these cell lines’ genomic DNAs at known proportions, and sequenced the mixture. We detected all variants present in this mixture, at allelic fractions tightly correlated with those expected given the cell lines’ mixing proportions (Pearson’s *r* = 0.998; Fig. 2). Based on samples of known VAF sequenced in replicate, the between-variant reliability of VAF estimated as an intraclass correlation coefficient was 0.998 (95% confidence interval 0.998 to 0.999) (see Supplementary Note).

**Table 1:** Genes targeted on smMIPs assay and proportion of CHIP in population covered by these target regions

Gene name	Target size (bp)	MIPS read depth		% of CHIP in Population^**b**^
		Median	Mean	
*ASXL1*	4687	2054	3891	7.41%
*CBL*	2803	4880	6845	0.62%
*DNMT3A*	3071	3163	4325	45.97%
*GNB1*	1023	4671	6139	0.97%
*PPM1D*	1894	3889	4856	4.01%
*SF3B1*	4045	3936	5073	2.46%
*TET2*	6165	2033	3587	19.13%
*TP53*	1714	4063	5543	1.97%
*U2AF1*	880	4403	6126	0.23%
*ZBTB33*	2019	1983	2632	2.22%
*ZNF318*	6918	3240	4765	1.80%
*SRSF2*^a^	1	289	512	1.88%
*IDH1*^a^	1	1325	1797	0.09%
*IDH2*^a^	1	3029	3909	0.30%
*JAK2*^a^	1	3839	4944	1.65%
**Overall**	**35,223 bp**	**2803**	**4480**	88.83%

^a^*SRSF2*, *IDH1*, *IDH2*, *JAK2* target a single hotspot mutation

^b^Population frequencies of CHIP previously reported in NHLBI TOPMed cohorts [6]

### CHIP prevalence in WHI samples

We applied our new CHIP sequencing assay to samples collected longitudinally from 182 subjects in the Women’s Health Initiative (mean: 3.0, range: 1–6 samples per subject; summarized in Tables S1 and S2). We obtained an overall median sequencing depth of 2803 (Table 1). After filtering, we detected a total of 206 CHIP driver mutations (defined as VAF ≥2%; Table S3). In a subset of 97 individuals who previously underwent both whole-genome sequencing (to ~30X depth) and deep smMIPS targeted sequencing (>1000X depth) of the same blood sample, 75/81 (92.3%) of the driver mutations called by WGS (mean VAF = 13.2%) were also detected by the smMIPS capture panel. The six mutations found by WGS but missed by smMIPS were in genes not included in the panel (*n* = 2), in a low coverage target *SRSF2* that was optimized in later captures (*n* = 2), or were long deletions that disrupted probe binding (*n* = 2 instances of a 23-bp *ASXL1* deletion). Despite the difference in sequencing depth between the two methods, we observed a correlation of 0.79 between VAFs measured by WGS and smMIPS, among driver mutations reaching VAF ≥ 2% by both methods (Fig. S1). Due to the deeper sequencing coverage, smMIPS sequencing detected an additional 103 driver mutations that went undetected by WGS; as expected, these tended to be at lower VAFs (mean VAF = 2.6%) for which the read depth provided by WGS (~ 30X coverage) is insufficient.

### Characteristics of CHIP mutations detected by smMIPS assay at initial WHI sampling

**Fig. 3:**
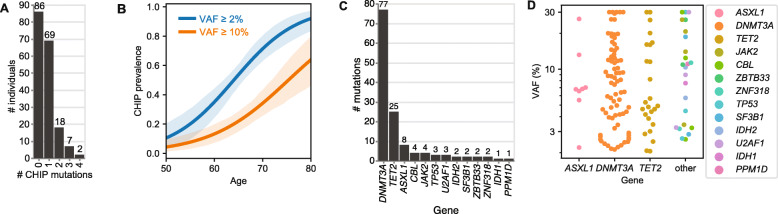
CHIP at initial blood draw. **A** Number of CHIP clones (driver mutations with VAF ≥ 2%) identified per subject at initial draw. **B** Prevalence of CHIP at VAF ≥2% (blue) or ≥ 10% (orange) by age at initial draw. **C** Number of mutations (VAF ≥ 2%) per gene. **D** Driver mutation VAFs grouped by gene

In the overall WHI sample (*N* = 182), at the initial timepoint sampled for each individual, 69/182 (38%) were CHIP-positive (carrying at least one driver mutation at VAF ≥2%), while 27/182 (15%) carried two or more such mutations (Fig. 3A). At the baseline timepoint, the most frequently mutated genes were *DNMT3A* (57% of driver mutations at VAF ≥2%), *TET2* (19%), and *ASXL1* (6%), consistent with prior WES or WGS reports [4, 6] (Fig. 3C). Among these were recurrent mutations at known hotspots including *DNMT3A* R882H/R882C (*n* = 8 individuals) and *JAK2* V617F (*n* = 10 individuals). In aggregate, clone sizes estimated by VAF were not significantly different by the gene mutated (Fig. 3D).

As expected, the prevalence of CHIP (defined as VAF ≥2%) at the initial blood sampling increased with age (Fig. 3B), from 18% in individuals with initial samples taken at age 60 years or younger, compared to 84% among individuals with initial samples taken at 70 years or older, with the high prevalence reflecting in part the selection criteria for subjects previously known to be CHIP-positive at baseline. In cross-sectional analyses, we observed a significant association between baseline BMI and age-adjusted CHIP VAF (*p*-value = 0.0446), but no association with other available baseline participant characteristics (race/ethnicity, smoking status) in this sample (Table S4). The BMI association is consistent with results from a WGS-based CHIP analysis showing the association of CHIP with obesity in a larger WHI sample [19].

### CHIP dynamics in longitudinal samples

**Fig. 4:**
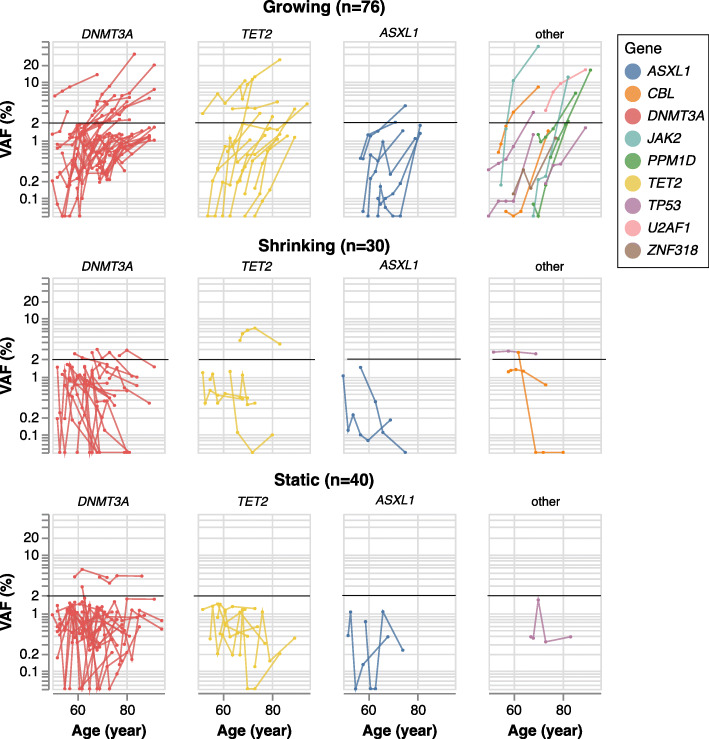
Longitudinal measurement of CHIP dynamics. Trajectories are shown, grouped by direction (rows) and gene (columns). Each trajectory corresponds to a single driver mutation in one subject, shaded by gene; black horizontal represents the VAF = 2% threshold

Of the 85 individuals with two or more blood draws for which no driver mutation at VAF ≥ 2% was detected in the initial sample, 49 (58%) developed at least one such driver mutation at the final sampling point an average of 13.9 years later. While these late-arising clones tended to remain small (only 11/49 reached VAF ≥ 10%), many were detectable above background at earlier time points even though they did not meet the working definition of CHIP. We classified the trajectories of each driver mutation, focusing on individuals (*n* = 65) for which there were three or more timepoints, with a VAF ≥ 1% clone in at least one of them. In these individuals, we identified 146 ‘trackable’ mutations, with 76 on growing trajectories, 30 shrinking, and 40 remaining static (Fig. 4). Among the mutations with a growing trajectory, the median rate of growth was 7.43 years (interquartile range: 4.48, 10.9 years) per doubling.

**Fig. 5:**
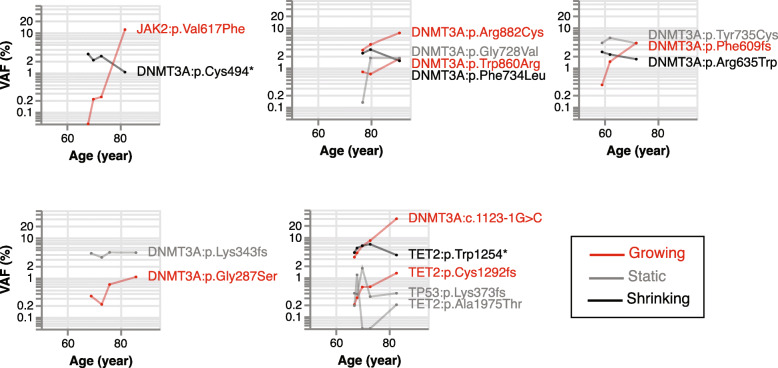
Competition among multiple different driver mutations in individual subjects. Each panel represents a single subject, and each driver mutation is a single line shaded by trajectory (red: growing, gray: static, black: shrinking)

Once mutations reached appreciable frequency, they tended to continue growing, indicating that after CHIP is acquired, it is generally not lost. Among the 76 growing trajectories, 34 (44.7%) reached the CHIP threshold of VAF ≥ 2% and 16 of these (21.1%) reached a VAF of ≥5%. By contrast, among the shrinking or static trajectories, only 10 and 2 mutations reached these respective VAF thresholds at any single timepoint. Of the 10 non-growing clones that reached VAF ≥ 2% at any timepoint, half involved another, growing trajectory detected in the same individual, and likely reflect competition from separate, fitter clones (Fig. 5).

### Growth rate varies by driver gene

We next examined driver mutation growth rate by CHIP gene and participant characteristics, as driver mutations in different CHIP genes may confer differential fitness advantages [20–23]. To examine this, we selected the dominant (largest VAF) trajectory from each individual, removing individuals whose dominant clone trajectories are shrinking or static, leading to 43 independent trajectories from 8 CH driver genes (Table 2). Due to the small number of clones for all but the 3 major CHIP driver genes (*DNMT3A, TET2, ASXL1*; *n* = 35 trajectories), we grouped trajectories for the other genes into a single “Other” category (*n* = 8). The rate of growth was higher among the “other” group, which included *CBL, JAK2, TP53*, and *U2AF1*, compared to the three major CHIP genes (*P* = 0.0013, Mann-Whitney U test). In addition, *DNMT3A* mutant clones were less likely to be in growing trajectories compared with other driver genes (OR = 0.52, *P* = 0.0085). Similar trends held among participants with only two timepoints, in which CHIP clones were classified as growing or non-growing. No association was observed between age-adjusted change in VAF (after log10 transformation) of dominant clones and any baseline participant characteristics (race/ethnicity, smoking status and BMI) in this sample (Table S5).

**Table 2:** Number of trajectories for each driver gene

Gene	*DNMT3A*	*TET2*	*ASXL1*	*CBL*	*JAK2*	*PPM1D*	*TP53*	*U2AF1*	Total
Growing, dominant	23	9	3	2	2	2	1	1	43
Growing, non-dominant	20	6	3	0	0	1	2	0	33
Shrinking	20	5	2	2	0	0	1	0	30
Static	30	7	2	0	0	0	1	0	40

## Discussion

Here we describe a rapid and cost-effective smMIPS-based assay that enables detection of CHIP in large scale longitudinal populations. Applying this assay to multi-time point samples from WHI participants demonstrates robust real-world performance in a large collection of longitudinal samples and reveals novel insights on clonal dynamics in a population without hematologic malignancy.

Most studies of CHIP to date rely upon either WGS or WES, or commercial capture kits which have high sequencing or library preparation costs, respectively, ranging from $150–$1000 per sample. The smMIPS approach offers a sensitive alternative at much lower per-sample cost ($30 per sample). Previous work using smMIPS for CHIP detection has been focused on individual hotspots [24] with full gene tiling of only *DNMT3A* [22]. Our results demonstrate that smMIPS can scale to fully tile gene sets which cumulatively account for nearly 90% of CHIP as determined by WGS.

Our application of the smMIPS assay to WHI reveals several important insights. We observe a significant burden of driver mutations below the conventional CHIP definition (VAF ≥ 2%) [4], enabled by deep sequencing coverage this assay provides. Indeed, for 97 individuals sequenced by both smMIPS and WGS, smMIPS detected 75 of the 81 driver mutations found by WGS, and an even greater number (*n* = 103) of driver mutations missed by WGS. Although these smMIPS-only clones tended to be less abundant, as expected, a subset nevertheless exceeded the working VAF ≥ 2% definition of CHIP. While these lower-VAF driver mutations may be less likely to have a clinical impact, in another WHI study, somatic mutations with any detectable VAF > 1% were associated with increased risk of acute myeloid leukemia [8]. Likewise, driver mutations in *DNMT3A* and *TET2* at frequencies as low as 1% have been associated with poor prognosis in chronic ischemic heart failure [25]. Thus, the clinical implications of these small clones (VAF range 0.1–2%) remain to be determined in future work, enabled through cost-efficient sequencing via assays like the one described here.

Our results add to recent observations regarding the longitudinal dynamics of clonal hematopoiesis suggesting driver gene-specific differences in clonal fitness. We find that CHIP clones detected among individuals without cancer do not inexorably grow: just over half of those observed did expand, with the remaining, mostly low-frequency clones divided roughly evenly between static and shrinking trajectories. Once mutations reached appreciable frequency, they tended to continue growing. Our results showing that *DNMT3A* mutant clones are less likely to be in growing trajectories are consistent with those of Fabre et al. [21] who found that clonal growth rate varies according to both age and driver gene mutation, with *DNMT3A* having a comparatively slow clonal growth rate in older aged adults. Similarly, using longitudinal targeted error-corrected sequence analysis in the Lothian Birth Cohorts, Robertson et al. [23] showed that clonal growth and fitness can differ substantially by gene, with splicing genes (such as *SF3B1*) having higher growth rates and clonal fitness compared to mutations in common genes such as *DNMT3A, TET2* or *ASXL1*.

In a longitudinal study of ultra-sensitive smMIP-based targeted gene sequencing of obese individuals, Van Deuren et al. [22] reported that metabolic factors such as insulin resistance and high density lipoprotein cholesterol may accelerate expansion of CHIP clones. While we did not detect any association of other baseline participant characteristics such as race/ethnicity, BMI, or smoking on clonal growth, larger sample sizes with serial sampling will be required to identify the genetic and environmental factors contributing to the differing outcomes of clonal competition and growth. This area of investigation has important clinical implications because mutations driving faster clonal growth, as reflected by a more rapid rise in VAF, carry a higher risk of malignant progression [21] and shorter time to development of AML [8].

Our study has several limitations. First, our assay robustly targets genes which account for ~ 90% of CHIP present in the population, so we may be misclassifying ~ 10% of CHIP-positive individuals due to omission of minor CHIP genes from the sequencing panel. This tradeoff was required to make the platform highly cost effective. However, a key benefit of the assay is that it is simple to extend to cover new targets, or to optimize coverage at existing ones, by spiking in new probes. In the present study, we leveraged this capability to add additional probes targeting a highly G + C-rich mutational hotspot in *SRSF2*, which increased its mean coverage from < 1 to 508. A second limitation of our study is that the availability of multi-time point samples was not uniform due to differences in the WHI study protocol. Third, there are other kinds of clonal hematopoiesis, such as mosaic chromosomal abnormalities (e.g. structural variants) that are not detected with our CHIP assay. These limitations are balanced by the significant strengths of the novel CHIP detection assay applied to one of the largest sample sizes studied to date.

## Conclusions

Our development of a novel smMIPS assay for CHIP detection enables scalable and cost-effective identification of CHIP in longitudinal multi-timepoint samples from WHI. This data enabled new observations on the spectrum of clonal hematopoiesis and clonal dynamics. Future investigations using this assay at scale may enable understanding of causes of these clonal dynamic phenomena and how changes in CHIP dynamics relate to diseases of aging associated with clonal hematopoiesis.

## Methods

### Samples

The Women’s Health Initiative (WHI) is a multicenter prospective study of risk factors for CVD, cancer, osteoporotic fractures, and other causes of morbidity and mortality among postmenopausal women [26]. Between 1993 and 1998, women aged 50–79 years from forty WHI clinical centers throughout the United States (US) were enrolled. All WHI participants completed a baseline screening visit at the time of enrollment which included blood sample collection. WHI participants have been followed prospectively for over 25 years. A subset of participants had blood collected at annual visits (AV) occurring at one, three, six, and 9 years after enrollment (AV1, AV3, AV6, AV9). An additional visit occurred between 2012 and 2013 (mean 15.4 years; range from 14 to 19 years after enrollment) as part of the WHI Long Life Study (LLS), which recruited a subset of 7875 surviving women ranging in age from 63 to 99 years at the time of LLS recruitment [27]. At each visit (baseline, AV1, AV3, AV6, AV9, LLS) genomic DNA was extracted from peripheral blood leukocytes using the 5 Prime DNA extraction kit.

A total of 182 WHI participants (without known prevalent hematological malignancy) were included in the current smMIPS-based sequencing study. These 182 individuals were selected either on the basis of either (a) having previously undergone WGS-based or targeted sequencing -based CHIP determination through the NHLBI TOPMed project (sample set A; *N* = 100) or having DNA samples at 3 or more time points (sample set B; *N* = 86). Sample set A was used to compare the detection of driver mutations between WGS and our new smMIPS capture panel. Therefore, we intentionally over-sampled WHI TOPMed participants who were previously determined to have CHIP (driver mutations at VAF ≥ 2% based on WGS or targeted sequencing) in order to directly compare intra-subject CHIP detection and VAF as determined by different assays using the same blood sample at the same time point. Sample set B was primarily to maximize our ability to assess longitudinal CHIP trajectories over time, and therefore includes mainly individuals who had DNA samples available at 4, 5 or 6 different time points. A detailed breakdown of number of samples at each time point (baseline, AV1, AV3, AV6, AV9, LLS) is provided in Table S1. Median age of participants was 62 years at baseline (range: 50–78 years) and 81 years (range: 66–95 years) at the LLS visit, respectively.

### Single molecule molecular inversion probe sequencing (smMIPS) assay

A smMIPS capture panel was designed to tile coding exons (+/− 5 bp) of the 11 most common CHIP genes [9] and recurrent mutational hotspots in four others (Table 1). Probe sequences were selected as previously described [28], with adjustments to eliminate the need for custom sequencing primers. Briefly, probe libraries were synthesized as a 12 k oligo pool by CustomArray (Bothell, WA) Inc., and subjected to bulk PCR amplification using flanking primers jklab0255_2019mipsPrep1f (GAGATCGGCGCGTTAGAAGAC) and jklab0256_2019mipsPrep1r (TGCAGGATCTAGGGCGAAGAC). PCR product was cleaned with 2.5X SPRI beads and eluted in 1X NEB cut smart buffer. To generate capture-ready probe pools, flanking adaptors were removed by BbsI-HF (#R3539L, NEB; Ipswitch, MA) digestion, overnight at 37 °C. Digested probes were cleaned by incubating with 1x volume SPRI beads (supplemented with 5 volumes isopropanol for 20 minutes), followed by washes in 70% ethanol and elution in Tris-EDTA pH 8. Poorly captured regions were tiled with additional probes (*N* = 112), synthesized as an oPool library by Integrated DNA Technologies (Coralville, IA) lacking flanking amplification adaptors and with 5′ phosphates. Original and make-up probes were combined into a single pool before use.

Capture reactions were assembled in a 96-well format, in 20 ul volume containing: probes (150:1 M excess to genomic DNA targets), 1X Ampligase buffer, 1 U Ampligase (Lucigen; Madison, WI), dNTPs at 0.4 uM each and 0.32 ul Hemo KlenTaq polymerase (NEB). Plates were incubated in a thermocycler at 95 °C for 10 minutes, 95 °C → 60 °C at − 0.1 °C/sec, followed by a hold at 60 °C for 18–24 hours. Exonuclease treatment was continued immediately after capture by adding 2 ul of mix containing 1X Ampligase buffer, 5 U Exonuclease I (NEB), and 25 U Exonuclease III (NEB) to each sample. Reactions were incubated at 37 °C for 45 minutes and 95 °C for 2 minutes. Dual indexed sequencing libraries were constructed by PCR amplification using indexing primers directed against common sequences on the probe backbone. Libraries were pooled at equal volumes, purified by 0.9X SPRI beads, and sequenced in batches of 196 on Hiseq 4000 or Novaseq instruments with paired-end 150-bp reads. Reagent, consumable, and sequencing costs total approximately $30 USD/sample.

Sequencing reads were aligned to the human reference genome (build 37) with bwa mem [29], and a custom sequencing pipeline (https://github.com/kitzmanlab/mimips) was used for post-alignment processing to remove probe arm sequences from each alignment and filter reads with duplicate unique molecular identifiers (UMIs).

### smMIPS assay validation and reliability

To validate the clone size detection limit of the smMIPS method, we prepared mixtures of gDNAs from five lymphoblastoid cell lines (GM06994, GM12878, GM20847, GM12877 and GM18507) with known genotypes, combined at 78.8, 16, 4, 1, 0.25%. Within the target region, these cell lines have 152 known variants as defined by the 1000 Genome Project (1000G) WGS genotypes and by detecting germline variants by sequencing cell lines individually. In the resulting mixture, their expected VAFs range from 0.125 to 100%. These variants constituted the true positive variant set. We also defined as ‘true negative’ sites 13 common polymorphism SNVs absent from all of the five cell lines, and those sites (+/− 50 bp) were defined as true negative variants. The positive control mixture was included with each sequencing batch for a total of 27 replicates. The between-variant reliability of VAF estimated as an intraclass correlation coefficient was 0.998 (95% confidence interval 0.998 to 0.999) (see Supplementary Note).

### Variant calling

Somatic SNPs and indels were called using LoFreq 2.1.3.1 [30], requiring minimum coverage 40, with ≥5 reads supporting the alternate allele and a variant allele frequency (VAF) ≥ 0.1%. Variants present in ≥5% of samples at a VAF of 1–10% were discarded as likely recurrent artifacts.

### CHIP calling

Variants were annotated using ANNOVAR software [31]. Variant calls processed using an existing filtering pipeline based upon gene name, variant functional class, and populational allele frequency [6]; workflow is available at available at https://app.terra.bio/#workspaces/terra-outreach/CHIP-Detection-Mutect2/notebooks. For *ZBTB33* and *ZNF318*, two genes not listed in [6], we included variants annotated as frameshift/splice-site/nonsense or nonsynonymous [32]. The full list of specific mutations queried is presented in Table S5. We manually reviewed alignments for selected CHIP variant calls using Integrative Genomics Viewer (IGV) [33].

### CHIP clone trajectories

To characterize the longitudinal trajectory of each CHIP clone over time, we restricted our analysis to individuals who (a) underwent smMIPS sequencing at least 3 time points and (b) had at least one driver mutation detectable at VAF > 1% at any of the timepoints. We excluded any variants with alternate read count < 2 or total read depth < 200. For each driver mutation meeting these criteria, we modeled the trajectory by fitting a linear regression: log10(VAF) = C + β * age; VAFs of zero were set to a minimum of 10^− 4^ (reflecting a conservative limit of detection for smMIPS), and each observation was weighted by the square root of the read depth. To further characterize clonal dynamics, we classified each trajectory based on linear trajectory, as (a) growing (β > 0, *P* < 0.5), (b) shrinking (β ≤ 0, *P* < 0.5), or (c) static (*P* ≥ 0.5). For trajectory analysis, we excluded CHIP clones with starting VAF > 10%, for which an exponential growth assumption may not fit.

### Association between participant characteristics and CHIP VAF and growth rate

For cross-sectional analyses at a single time point, we fit linear and logistic regression models to assess the relationship of either CHIP prevalence (total clones or large clones only) or log-transformed VAF to age at blood draw, race/ethnicity, smoking status, or BMI. We used the first visit time point for each subject. To assess the relationship of these same participant characteristics, to clone growth over time, we utilized longitudinal data from all individuals with sequencing data from at least two time points with positive VAF observations (*N* = 148 individuals). For each participant, we first selected a single driver clone, prioritizing as the predominant clone those with the highest VAF at any follow-up timepoint. We used a linear regression to determine the effect of age, race/ethnicity, smoking, or BMI on the difference (after log10 transformation) between the first non-zero VAF value and the last non-zero VAF. All statistical analyses were adjusted for the first visit time of year and performed in R version 4.2 (R Core Team, URL https://www.R-project.org/).

## Supplementary Information

**Additional file 1.**

**Additional file 2.**

## Data Availability

Sequencing data underlying this study is deposited in dbGaP (phs000200.v12.p3).
